# Altered Patterns of Maternal Behavior Transitions in Rats Exposed to Limited Bedding and Nesting Material Paradigm

**DOI:** 10.1002/brb3.70113

**Published:** 2024-10-23

**Authors:** Grace E. Pardo, Lucero B. Cuevas, Luis F. Pacheco‐Otalora, Enver M. Oruro

**Affiliations:** ^1^ Neuroscience Research Laboratory, Scientific Research Institute Andean University of Cusco Cuzco Peru; ^2^ Neurocomputing, Social Simulation and Complex Systems Laboratory, Scientific Research Institute Andean University of Cusco Cuzco Peru

**Keywords:** attachment, development, maternal care, network analysis, nursing behavior, sensitive periods

## Abstract

**Introduction:**

Maternal care plays a fundamental role in early life, and the alteration of its patterns can negatively affect the developmental course of the offspring in a myriad of domains in both rats and humans. The limited bedding and nesting (LBN) protocol is an extensively used paradigm in rodents to address the impact of altered maternal behavior patterns on infants' neurodevelopment. Here, we explore the altered patterns of maternal care in rats in LBN conditions by describing sequences of transition between maternal behavior components using network analysis. Using this technique, we capture how often maternal behavior transitions take place during the LBN period and which behaviors play central roles in those transitions over time.

**Materials and Methods:**

Female rats and their pups were placed in standard and LBN housing conditions from Postpartum Days 2 to 9, during which maternal behavior was observed during the light and dark phases. We used inferential statistical analysis to compare the maternal behavior profiles of control and LBN dams, and network analysis was used to capture the altered sequence of maternal behavior transitions during the period of LBN.

**Results:**

Compared to control dams, LBN dams significantly increased their high crouch nursing posture during light/dark phases (*p *= 0.018), and the number of behavioral transitions increased only during the dark phase (*p *= 0.0004). Network analysis revealed specific altered patterns of behavioral transitions in LBN dams, characterized by the predominance of switches between active nursing postures during the first five days of the LBN protocol.

**Conclusion:**

Nursing behavior was the most disrupted component of maternal behavior under the LBN protocol, mainly during the dark phase. Network analysis can complement and extend traditional methods to gain a more thorough understanding of maternal care strategies and behavioral patterns in LBN conditions and potential consequences for the offspring.

## Graphical abstract

The limited bedding and nesting (LBN) protocol affects rat maternal behavior by increasing high crouch posture, reducing exits from the nest, and increasing behavioral transitions. Further network analysis shows that LBN primarily disrupts high crouch nursing (HG) posture and nest exits (OFF‐nest) patterns of dams during early postpartum days.

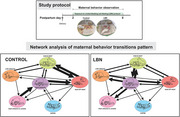

## 1 Introduction

A large body of empirical work has shown that maternal care plays a paramount role in early life, determining the trajectories of offspring development that predict outcomes in many domains, including physical, emotional, cognitive, and social–behavioral domains in humans and animals (for review, see Curley and Champagne 2016; Ferreira de Sá, Camarini, and Suchecki 2023; Glynn and Baram 2019; Sullivan and Opendak 2020). The altered patterns of maternal care appear to be the primary means by which mothers influence the ongoing development of their infant's biological systems (Dozier et al. 2023; Lee et al. 2019; Rifkin‐Graboi et al. 2015; Davis et al. 2017; Davis et al. 2019; Granger et al. 2021; Holmberg et al. 2022; Noroña‐Zhou et al. 2020), which has also been shown in rodent studies, mainly using the limited bedding and nesting (LBN) paradigm. This paradigm mimics the early‐life experience of an impoverished home environment. It is achieved by placing the rodent dams and their pups in restricted bedding and nesting material and wire mesh flooring home cages during early postnatal days (Ivy et al. 2008; Pardo et al. 2023; Rice et al. 2008; Walker et al. 2017). In this condition, the dam's patterns of maternal care become fragmented, which reduces the predictability of maternal‐driven sensory stimulus for the pup's neurobehavioral development processes in the short and long terms (Ivy et al. 2008; McLaughlin et al. 2016; Shupe and Clinton 2021).

In the LBN condition, maternal care patterns have been documented in several studies in rats (Davis et al. 2017; Granata et al. 2021; Ivy et al. 2008; Molet et al. 2016; Fuentes et al. 2014; Shupe and Clinton 2021) and mice (Pardo et al. 2023; Rice et al. 2008). Fragmentation of maternal care patterns could be quantified by measuring the number of transitions between discrete maternal behavior components (Ivy et al. 2008; McLaughlin et al. 2016; Shupe and Clinton 2021), which has been proposed as a representative measure of the total maternal care pattern possible to be associated with infant development outcomes (Fuentes et al. 2014; Granata et al. 2021). Although this analysis of transitions effectively characterizes an altered or fragmented pattern of total maternal behavior in the LBN condition, there is a lack of understanding of how these transitions occur across the different components of maternal care from day to day during each observation period. Exploring the qualitative aspect of maternal behavior transition patterns in LBN conditions could give us a better appreciation of the mother–infant interaction during early life and improve our understanding of its impact on pups' development.

To capture the qualitative changes in maternal behavior transition, we introduce a network analysis method in addition to the traditional statistical analysis of frequencies. Network analysis is a commonly used method to capture and represent complex and rapidly changing behavior across multiple time scales in patterns of connections in specific social networks (Wasserman and Faust 1994). However, in recent years, this method has started to be applied to capture patterns within an individual's behavior of human infant development (DiMercurio et al. 2018; Thurman and Corbetta 2020). In this study, we explore the alterations in maternal behavior of rats exposed to the LBN paradigm, focusing on how individual behavior changes over time and describing the pattern of specific sequences of maternal behavioral component transitions throughout the postpartum days (PPDs) in the light and dark phases utilizing network analysis. To the best of our knowledge, this is the first study using this methodological tool to analyze individual maternal behavior changes.

## 2 Materials and Methods

### 2.1 Animals

Virgin 2–3 months old Sprague Dawley rats (Charles River, USA) were mated at the Andean University of Cusco animal facility to produce a total of 21 litters. The animals were group‐housed in a 30 × 30 × 18 cm cage, with four animals per cage. The facility operated on a 12‐h light/dark cycle schedule (light on at 6 a.m.) and maintained temperature (22–23°C) and humidity (45%). Food and water were provided ad libitum. Animals were mated in the house and on Gestational Days 15–18. Experiments involved two cohorts containing 10 and 11 litters each. On Gestational Days 15–18, dams were placed in individual cages and checked daily at 5 p.m. for parturition, with the day of birth considered as PPD 0. All experimental procedures followed the Guidelines for Animal Care and Use of Laboratory Animals of the National Institutes of Health (2011) and were approved by the Institutional Animal Care and Use Committee of the Andean University of Cusco (approval number N°002‐2022‐CIEI‐UAC).

### 2.2 LBN Protocol

On PPD 2, the litter size was culled to eight to nine pups with an equal number of males and females when possible. Pups were cross‐fostered among litters born on the same day and with similar body weights to provide an equal number and sex distribution in each litter. As described in previous studies, the pups of each litter were weighed and randomly assigned to control or LBN housing conditions (Ivy et al. 2008). Control dams were placed in standard home cages with 2500 mL of woodchip bedding and nesting material (two pieces of paper towel). The LBN dams were placed in a standard cage with wire mesh, 0.8 × 0.8 cm opening, placed 2 cm above the cage floor with woodchip under the metal grid to absorb excess ammonia with one piece of paper towel as nesting material. Both housing conditions were left undisturbed until PPD 10 in the morning, after which all dams and their pups were returned to standard housing conditions, where they remained until weaning.

### 2.3 Maternal Behavior Observation

From PPD 2 to 9, maternal behavior was observed in the home cage three times a day for 72 min each time, using a protocol adapted from our previous work (Pardo et al. 2016). The observation was made on dams from both cohorts (*n* = 10 and 11 litters per condition; one control litter and two LBN litters were excluded from the analysis due to low litter size). Each cage was observed twice during the light phase (at 9:00 a.m. and 2:00 p.m.) and once during the dark phase (at 6:00 p.m.). The dark phase observations were conducted under red light. These observation periods were chosen to represent the maternal behavioral profile in the day and dark phases based on a previous observation made by our group (Pardo et al. 2016). Within each observation period, the behavior of each dam was scored every 3 min resulting in 25 observation per period for a total 75 observations per 24 h cycle for the following maternal behavioral repertoire, which are considered the most relevant maternal behavior that rat exhibit during postpartum period (Champagne et al. 2003; Rosenblatt 1969): high crouch posture (HG, mother nursing pups in an arched‐back posture); low crouch posture (LW, mother nursing pups in a “blanket” low arched back posture); supine posture (SUP, a passive posture in which the mother is lying on her back or side while the pups nurse); licking the pups (L, licking/grooming the surface of their bodies and their anogenital regions); HG posture at the same time the mother licking the surface of pup' bodies and their anogenital regions (HG/L); dam in the nest (DN, dam in the nest area, mostly besides the pups, without showing any nursing behavior); and mother off the nest (OFF, the lactating female is out of the nest). During the 9:00 a.m. observation period on PPD 2 and PPD 10, maternal behavior was observed at least 30 min after the dams, and litters were transferred to new cages (LBN and control housing conditions) to allow the dam to acclimate to the new environment. The analysis considered the number of events of each maternal behavioral component. Moreover, we analyzed maternal behavior by considering the sum of transitions in each behavioral component in the light and dark phases for quantitative analysis. The profile of the behavioral components observed at 9:00 a.m. for the control group was not statistically different from those observed at 2:00 p.m., so both periods were averaged to represent the light phase data (see Table S2). Data are reported as the number of observation events in the light (average of the two observation periods) and dark phases and were averaged among the females in each group.

### 2.4 Statistical Analysis

Statistical analyses were performed using GraphPad Prism 9.1.2 software (GraphPad, San Diego, CS, USA). All data are expressed as mean ± SEM, and the statistical significance level was considered when *p* < 0.05. Two‐way repeated measure (RM) ANOVA followed by Šídák's multiple comparisons post hoc test was used to analyze maternal behavior components. Asterisks denoted significant results for significant interactions for two‐way ANOVA in associated figures.

### 2.5 Network Analysis

**FIGURE 1:**
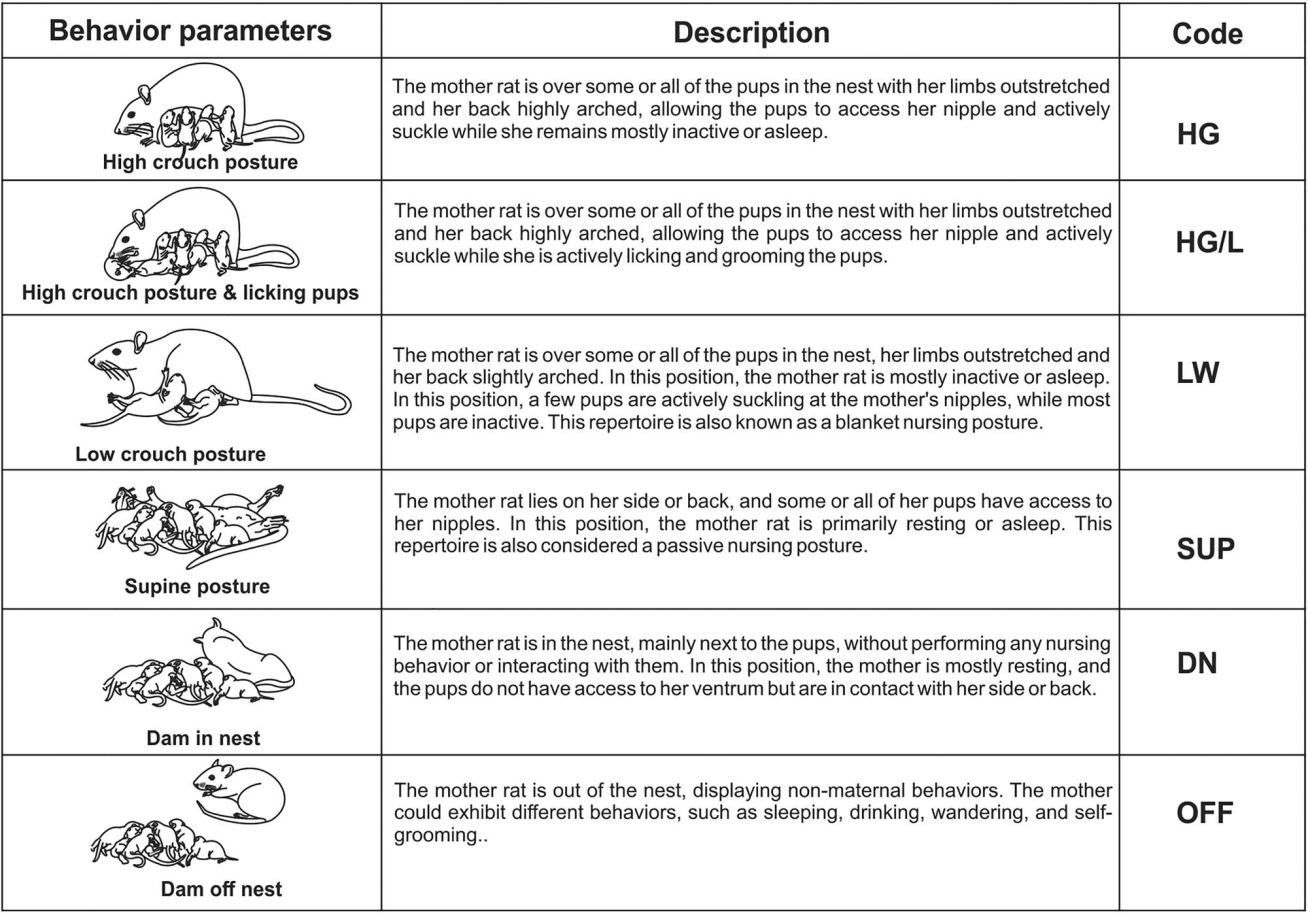
Maternal behavior repertories of rats considered for network analysis. Depiction of the different components of maternal behavior exhibited by the mother rat during postpartum days (Moore and Chadwick‐dias 1986; Myers et al. 1989; Rees, Lovic, and Fleming 2004; Rosenblatt 1969). The syntaxis used for each component in network construction is added.

**FIGURE 2:**
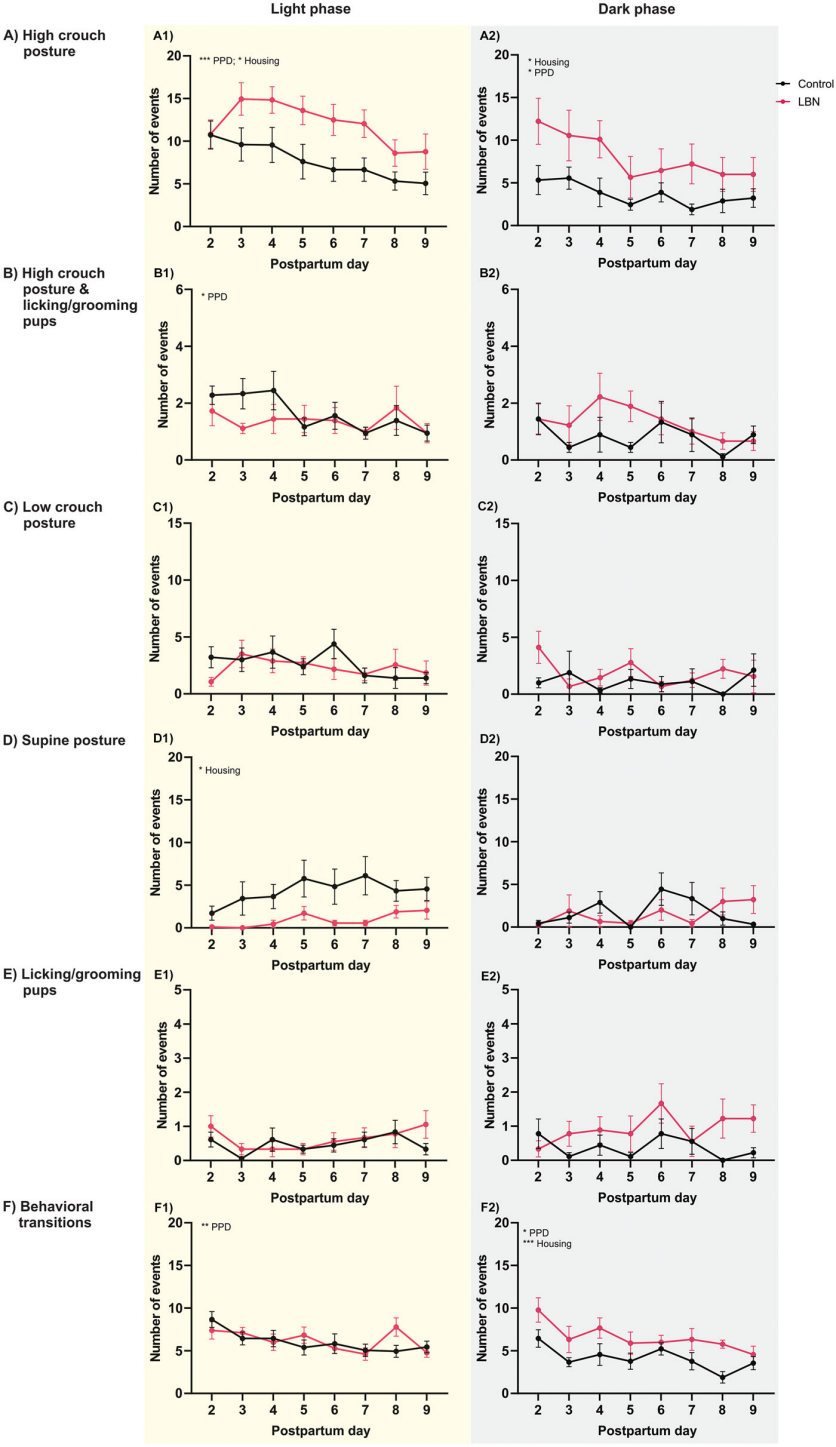
Maternal behavior profile during LBN protocol. During the postpartum, the high crouch posture of dams (A) increased significantly in the LBN condition compared to control dams in light (A1) and dark phases (A2). High crouch posture and licking components (B) showed a significant reduction as a function of postpartum days only during the light phase (B1) but not in the dark phase (B2). Low crouch posture (C) did not show changes along the postpartum days, nor was it affected by LBN in the light phase (C1) or dark phase (C2). Supine posture (D) was significantly reduced in LBN dams compared to control during the light (D1) but not in the dark phase (D2). Licking/grooming pups (E) did not show changes along the postpartum days, nor was it affected by LBN in light (E1) or dark phase (E2). Behavioral transitions (F) were significantly reduced as a function of postpartum days during the light phase (F1). A similar tendency was found in the dark phase in LBN dams compared to controls (F2). Data are mean ± SEM. *p < 0.05, **p < 0.01, ***p < 0.001, ****p < 0.0001. *N* = 9 per group.

Network analysis was performed to explore the changes in the pattern of specific maternal behavioral sequence transitions along PPDs in control and LBN dams. We expected these sequences or transitions to be expressed as a network represented by nodes and links. For the network analysis, we used data from nine dams of each group (control and LBN), the same that was used for statistical analysis. Nodes represent maternal behavioral parameters, whereas links represent the connection between two nodes. In this analysis, the nodes were generated by each maternal behavioral parameter. A history of transitions between nodes was determined as an average of nine dams per observation phase, and each transition was treated as a directed link between any two nodes. We did not presume any self‐links in the network analysis. Data from the 9:00 a.m. and 2:00 p.m. observations were averaged for the light phase network. A node was generated where a particular maternal behavioral component occurred. In the present analysis, the following six maternal behavior components were picked as representative nodes based on the most frequent occurrence during the PPDs: HG, LW, SUP, HG/L, DN, and OFF (Figure 1). These specific behaviors were selected as nodes because they directly involve physical mother–infant contact, which is crucial for the purpose of this work. The component licking/grooming the pups was not considered in the network construction because it occurred very little alone (see Figure 2F) and consistently in conjunction with another behavior component of the dam, generally during HG nursing posture, which was considered a node in the network as HG/L node.

Considering that our data collected were the number of behavioral events in individual animals throughout continuous sampling observation and the behavior diffusion had only one direction, we constructed a directed and weighted network. Figure S1 illustrates the raw data of maternal behavior observation and the graph from which it was converted. Networks were constructed and visualized using NetLogo 6.2.2 (Wilensky 1999). Each of the six maternal behavior components was used as a node in the network and is represented by different colors. The size of each node indicates the number of times each component occurred within a period of observation. The connection of nodes was described using arrows and links, and their thickness indicates the direction and frequency of transitions between pairs of nodes. The formulae and code of the networks are available at https://sites.google.com/view/orurolab/behavioraldata.

The following measures characterized the network structures of control and LBN: (1) *in‐strength*, (2) *out‐strength*, and (3) *betweenness centrality*. In‐strength and out‐strength measures show how nodes were connected with the most incoming connections from other nodes and outgoing connections to other nodes, respectively. These were calculated from the total weight of each link. The in‐strength measure was used to determine how strongly a behavioral component is influenced by other components, whereas the out‐strength measure determined how strongly a behavioral component influenced other components. The betweenness centrality shows the number of times a given node lies on one of the paths between all pairs of nodes in the network (Wasserman and Faust 1994), which was used to capture the importance of a given maternal behavior component as a bridge that the mother rats use to transition to other behavioral components. Centrality measures are presented as standardized values (*z*‐scores).

## 3 Results

Sprague Dawley rats were exposed to the LBN protocol from PPD 2 to PPD 9, and maternal behavior observations were conducted at three moments of the day (two during the light phase and one during the dark phase). The statistical analysis described in Section 3.1 showed that the LBN condition induces alterations in maternal care by increasing HG nursing postures during both phases, reducing SUP nursing postures in the light phase, reducing the exits from the nest, and increasing behavioral transitions in the dark phase. The specific alterations in maternal behavior transition patterns during LBN were found by network analysis, as described in Section 3.2.

### 3.1 Changes of Maternal Behavior During LBN Protocol

The data from the light phase (9:00 a.m. and 2:00 p.m.) were averaged and analyzed as the profile of the light phase, and data from 6:00 p.m. were analyzed as the profile of the dark phase (see Figures 2 and S2). The data on maternal behavior were analyzed using a two‐way RM ANOVA analysis, and the results are detailed in Tables 1 and S1.

**TABLE 1:** Summary of two‐way repeated measure ANOVA results on dam behaviors as a function of postpartum day and housing condition (control and LBN).

Parameters	Factors				Interaction	
	Postpartum day		Housing		Postpartum day × housing	
	*F* (7,112)	*p*	*F* (1,16)	*p*	*F* (7,112)	*p*
Light phase						
High crouch posture	5.054	**< 0.0001**	7.004	**0.0176**	1.150	0.3372
Low crouch posture	1.299	0.2571	0.253	0.6218	1.004	0.4322
Supine posture	1.802	0.0938	8.134	**0.0115**	0.803	0.5861
High crouch posture and licking	2.178	**0.0414**	0.489	0.4941	1.210	0.3034
Licking/grooming pups	1.920	**0.0727**	0.4986	0.4903	0.927	0.4881
Behavioral transitions	3.432	**0.0023**	0.095	0.7622	1.568	0.1521
Dark phase						
High crouch posture	2.564	**0.0173**	6.917	**0.0182**	0.538	0.8041
Low crouch posture	0.748	0.6317	3.115	0.0966	1.050	0.4009
Supine posture	1.503	0.1733	0.090	0.7677	1.871	0.0809
High crouch posture + licking	1.635	0.1328	1.469	0.2431	1.011	0.4276
Licking/grooming pups	1.101	0.3672	3.554	0.0777	1.323	0.2458
Behavioral transitions	3.364	**0.0027**	20.06	**0.0004**	0.553	0.7627

*Note*: Table shows a two‐way RM ANOVA analysis of maternal behavior parameters with postpartum days and housing condition (control and LBN) as main factors. Bold text denotes *p* < 0.05.

The analysis of HG events during the light phase showed a significant reduction as a function of PPDs (*p* < 0.0001). It changed with housing condition (*p* < 0.05) with no interaction, where LBN dams had an increased number of high crouch (HG) posture events than control dams (Figure 2A1). During the dark phase, HG posture events also reduced throughout the PPDs (*p* < 0.05), with changes in housing condition (*p* < 0.05) and no interaction, where LBN dams showed increased HG posture events compared to control dams (Figure 2A2). An analysis of the combination of HG nursing posture with licking and grooming pups (HG/L) during the light phase showed a significant reduction throughout PPDs (*p* < 0.05) with no effect on housing conditions (Figure 2B1). No effect of PPDs or housing conditions was found for HG/L during the dark phase (Figure 2B2). Analysis of low crouch (LW) nursing posture at the light or dark phase showed no change with PPDs or housing conditions (Figure 2C1,C2). The analysis of supine (SUP) nursing posture in the light phase showed no change along the PPDs. However, it changed with housing conditions (*p* < 0.05), where LBN dams showed significantly decreased SUP posture events compared to control dams (Figure 2D1). During the dark phase, analysis of SUP posture showed no change in PPDs or housing conditions (Figure 2D2). There was no effect of PPDs or housing conditions on licking/grooming pups (L/G) during the light (Figure 2E1) or dark phase (Figure 2E2).

The two‐way ANOVA analysis of behavioral transition showed a significant effect of PPDs during the light (Figure 2F1) and dark phases (Figure 2F2), where transition reduced as PPDs advanced (*p* < 0.01), and only during the dark phase this transition changed with housing condition (*p* < 0.001), where LBN dams showed increased transition events compared to control dams.

The non‐pup‐directed behaviors were also analyzed and are reported in Table S1 and Figure S2. DN component analysis during the light phase showed a significant main effect of PPDs (*p* < 0.001) but no significant effect on housing conditions (Figure S2A1). There was no impact of PPDs or housing conditions on DN during the dark phase (Figure S2A2). The analysis of the dam OFF‐nest event increased significantly along the PPDs during the light phase (*p* < 0.0001) but did not change with housing conditions (Figure S2B1). During the dark phase, the OFF‐nest event also significantly increased along the PPDs (*p* < 0.01). It changed with housing condition (*p* < 0.001), with no interaction (*p* < 0.05), where LBN dams showed reduced OFF‐nest events compared to control dams (Figure S2B2). The analysis of the number of events the pups were off‐nest (pup off) showed no effect of PPDs (*p* > 0.05) or housing conditions during the light phase (Figure S2C1), and similar results were found during the dark phase (Figure S2C2). During the light phase, the analysis of pup recovery events showed a significant main effect of PPDs (*p* < 0.0001), housing conditions (*p* < 0.05), and interaction (*p* < 0.01). A Šídák's multiple comparisons test showed a significant increase of pup recovery events in LBN dams on PPD 2 compared to control dams (*p* < 0.0001) (Figure S2D1).

No effect of PPDs or housing conditions was found for pup recovery during the dark phase (Figure S2D2). Analysis of the number of nest‐building behavior events during the light phase showed a significant effect of PPDs (*p* < 0.0001) with no effect of housing conditions but significant interaction (*p* < 0.0001). A Šídák's multiple comparisons test showed a significant increase of nest‐building events in LBN dams on PPD 2 compared to the control dams (*p* < 0.0001) (Figure S2E1). Similarly, nest‐building events showed a significant effect of PPDs (*p* < 0.01) and housing conditions (*p* < 0.01) with no interaction between the two factors (*p* = 0.05) during the dark phase (Figure S2E2). Finally, no effect of the PPDs factor or housing conditions was found for self‐grooming behavior during the light (Figure S2F1) nor the dark phase (Figure S2F2).

### 3.2 Network Analysis of Maternal Behavior Transitions During the LBN

**FIGURE 3:**
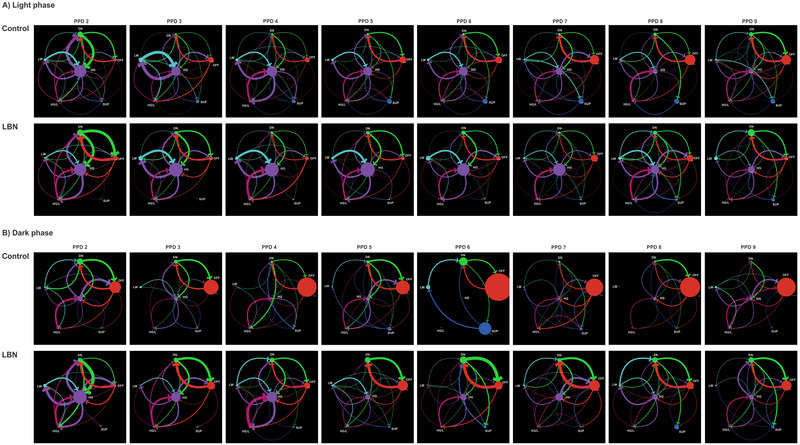
Visualization of temporal changes in the maternal behavior transition networks from PPD 2 to PPD 9 in control and LBN dams. Control and LBN maternal behavior transition network during the light (A) and dark phases (B). The graph of each network was generated with six nodes. Each node's size represents the average number of events in each observation period (light and dark phases). Links connect each pair of nodes. Bolder lines indicate a stronger connection between nodes. Each network was generated by the average maternal behavior data corresponding to nine rats per group for light (average of 9:00 a.m. and 2:00 p.m. observation) and dark phases (6:00 p.m. observation). Graphs were generated using NetLogo software. DN, dam on the nest; HG, high crouch nursing posture; HG/L, high crouch nursing posture and licking pups; LW, low crouch nursing posture; OFF, dam off nest; SUP, supine nursing posture.

We conducted a network analysis to investigate the changes in the patterns of transitions between different components of maternal behavior in the LBN condition. Unlike traditional statistical analysis, network analysis allowed us to visually capture the qualitative changes in maternal behavior transitions during the PPDs. Figure 3 shows the network structure depicting the connection between nodes and the size of each node. The size of each node is based on the frequency of appearance of each behavioral component during each observation phase. For example, in Figure S1, node OFF (red) is the largest node in the network, representing a much more frequent behavioral component.

During the light phase, the network of control dams had a larger HG node size from PPD 2 to 6, with the OFF node being the largest after this period. On the other hand, the network of LBN dams had a larger HG node size from PPD 2 to 9. This suggests that control dams showed more of the HG component during the first few days after birth and more of the OFF component later, whereas LBN dams exhibited primarily the HG component throughout all observation days (Figure 3A). During the dark phase, the network of control dams had a larger OFF node size from PPD 2 to 9, whereas networks of LBN dams had larger HG nodes from PPD 2 to 4, with the OFF node being the largest after this period. This suggests that control dams exhibited primarily the OFF component throughout all observation days, whereas LBN dams showed more of the HG component during the early days of postpartum and more of the OFF components later (Figure 3B).

The size of the network nodes in Figure 3 captures an integrated visualization of the data presented in Figure 2 using a typical statistical analysis of the frequency of events for each component of maternal behavior.

#### 3.2.1 Out‐Strength and In‐Strength Centrality

**FIGURE 4:**
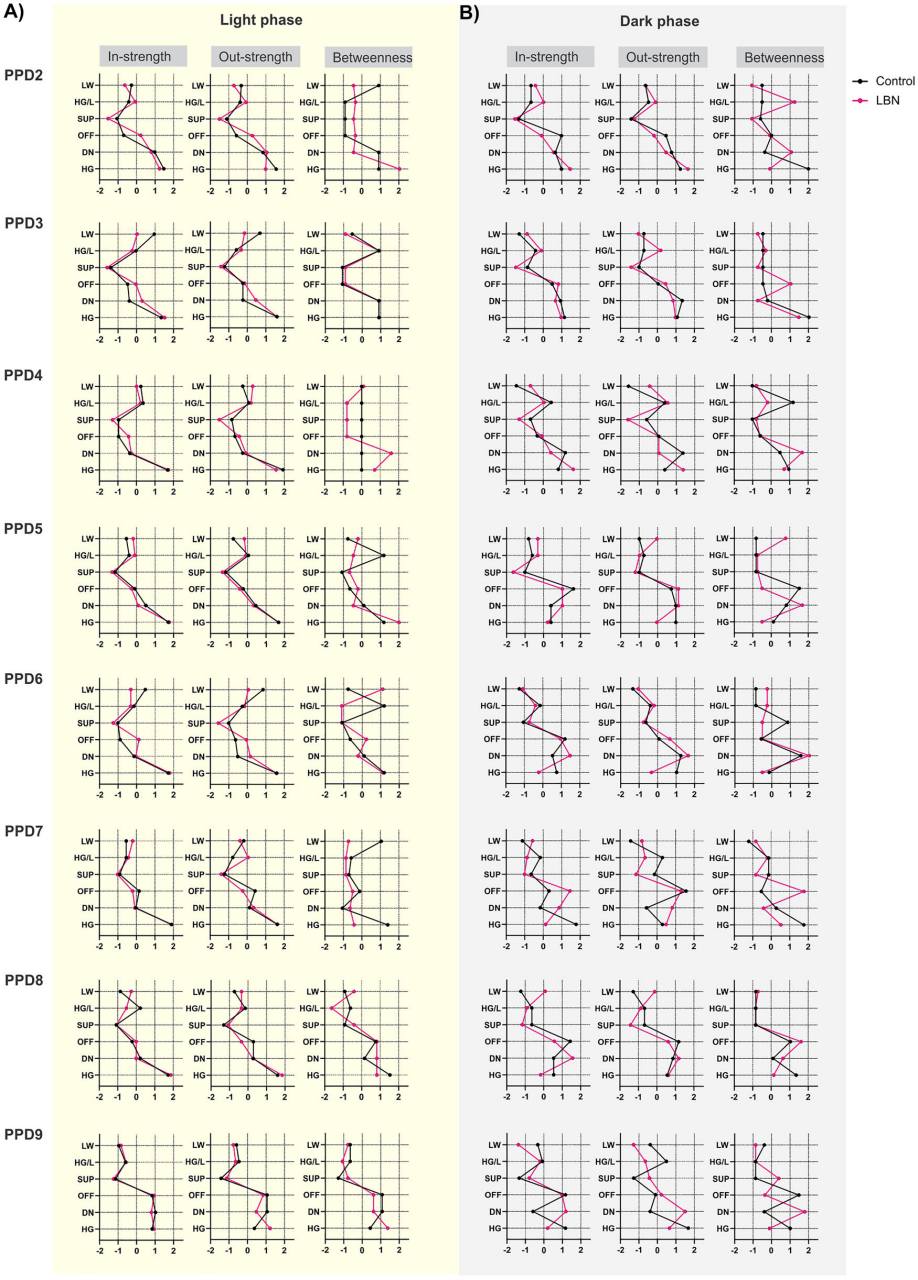
Centrality measures for the control and LBN through postpartum days. The figures show the in‐strength, out‐strength, and betweenness centrality depicted in columns for each postpartum day for the light phase (A) and the dark phase (B). The black line represents the control networks and the magenta line represents the LBN networks. Values shown on the *x*‐axis of the figures are standardized *z*‐scores. The most influential nodes are those with centrality valued above zero. DN, dam on the nest; HG, high crouch nursing posture; HG/L, high crouch nursing posture and licking pups; LW, low crouch nursing posture; OFF, dam off nest; SUP, supine nursing posture.

Centrality plots for out‐strength and in‐strength are presented in Figure 4; both measures were computed to investigate which nodes (behavioral components of maternal behavior) were most influential in generating a change in the maternal behavior pattern across the PPDs. Results of these measures indicated that in the light phase, both control and LBN dams exhibited similar maternal behavior patterns sustained by DN and LW. However, this pattern differed during the dark phase. Control dams initially relied on the OFF and DN components and then primarily on the OFF components. In contrast, LBN dams showed more reliance on the HG component initially and then on DN and OFF components.

During the light phase, HG, followed by DN and LW, had higher scores for in‐strength centrality across the PPDs (Figure 4, light phase). Differences between control and LBN networks were only on LW, in which the LBN network scored lower on PPD 3 and 6. The behavioral components with higher out‐strength scores across the PPDs were the same as those with higher in‐strength centrality, and differences between networks were noticeable only on LW, in which the LBN network scored lower on PDD 3 and 6. The SUP and OFF were the least influenced by other components, as indicated by the lowest in‐strength and out‐strength indexes, except on the last day, where OFF scored higher in both centralities (Figure 4, dark phase). This means that HG, followed by DN, and, to a lesser extent, LW were the components most strongly influenced by other components that influenced the temporal changes in maternal behavior across the PPDs.

During the dark phase, OFF, followed by HG and DN, scored higher on in‐strength across the PPDs (Figure 4, dark phase). There were notable differences between the control and LBN networks across the PPDs. OFF scored lower on PPD 2 and 8, but higher on PPD 7, whereas HG scored higher on PPD 2 and 4 but lower on PPD 6, 7, 8, and 9. DN scored lower on PPD 4 but higher on PPD 7, 8, and 9. The components with higher out‐strength scores across the PPDs were HG, followed by DN and OFF (Figure 4, dark phase). Differences between control and LBN networks were notable across PPDs. HG scored higher on PPD 2 and 4 but lower on PPD 5, 6, and 9. DN scored lower on PPD 4 but higher on PPD 7 and 9. OFF scored lower on PPD 2. LW, SUP, and HG/L were less influenced by other components, as indicated by the lowest in‐strength and out‐strength indexes. This indicates that in the dark phase, maternal behavior patterns of control and LBN dams were sustained by different behavioral components over the PPDs. From PPD 2 to 4, the control dam's behavior was mainly sustained by the OFF and DN components, whereas the behavior of LBN dams relied more on the HG component. Between PPD 5 and 9, the control dam's behavior was mostly sustained by the OFF component and less by the HG and DN components. In contrast, the behavior of LBN dams during this period relied more on DN, OFF, and HG components. These centrality measures also reflect the behavioral components reported using traditional frequency measures (Figure 2), but network analysis also tells us which components the dams used most frequently to transition to other components.

#### 3.2.2 Betweenness Centrality

Central measurement analysis was performed to determine whether some nodes are more central than others in the network. The node with the highest betweenness centrality in the network indicates that it is the component that dams use most to transition to other behaviors during the observation period. There were differences between control and LBN patterns during the light and dark phases, especially in the early PPDs. During the light phase, the behavioral component that LBN dams used most to transition to other behaviors was HG, and during the dark phase, it was DN, which was different from control dams that used most HG in both phases.

In the light phase, the HG node, followed by DN, HG/L, and LW nodes, scored higher on betweenness centrality across PPDs (Figure 4, light phase). Differences between the control and LBN networks were notable on different PPDs. The HG node scored higher in betweenness centrality on PPD 2, 4, 5, and 9 in the LBN network but lower on PPD 7 and 8 than the control. The DN node scored higher on PPD 4 and 8 but lower on PPD 2 and 9. The HG/L node scored lower on PPD 5 and 6. The LW node scored higher in centrality on PPD 6 but lower on PPD 2 and 7. The OFF node scored lower on PPD 9.

During the dark phase, the HG node, followed by DN, OFF, SUP, and HG/L nodes, had higher betweenness centrality scores across PPDs (Figure 4, dark phase). Differences between control and LBN networks were evident on different PPDs. Specifically, the HG node scored lower on PPD 2, 7, 8, and 9, whereas the DN node scored higher on PPD 2, 4, 8, and 9. The OFF node had higher scores on PPD 3, 7, and 8 but lower on PPD 5 and PPD 9. In addition, the SUP node scored lower on PPD 6 but higher on PPD 9, and the HG/L node scored higher on PPD 2 but lower on PPD 4.

**FIGURE 5:**
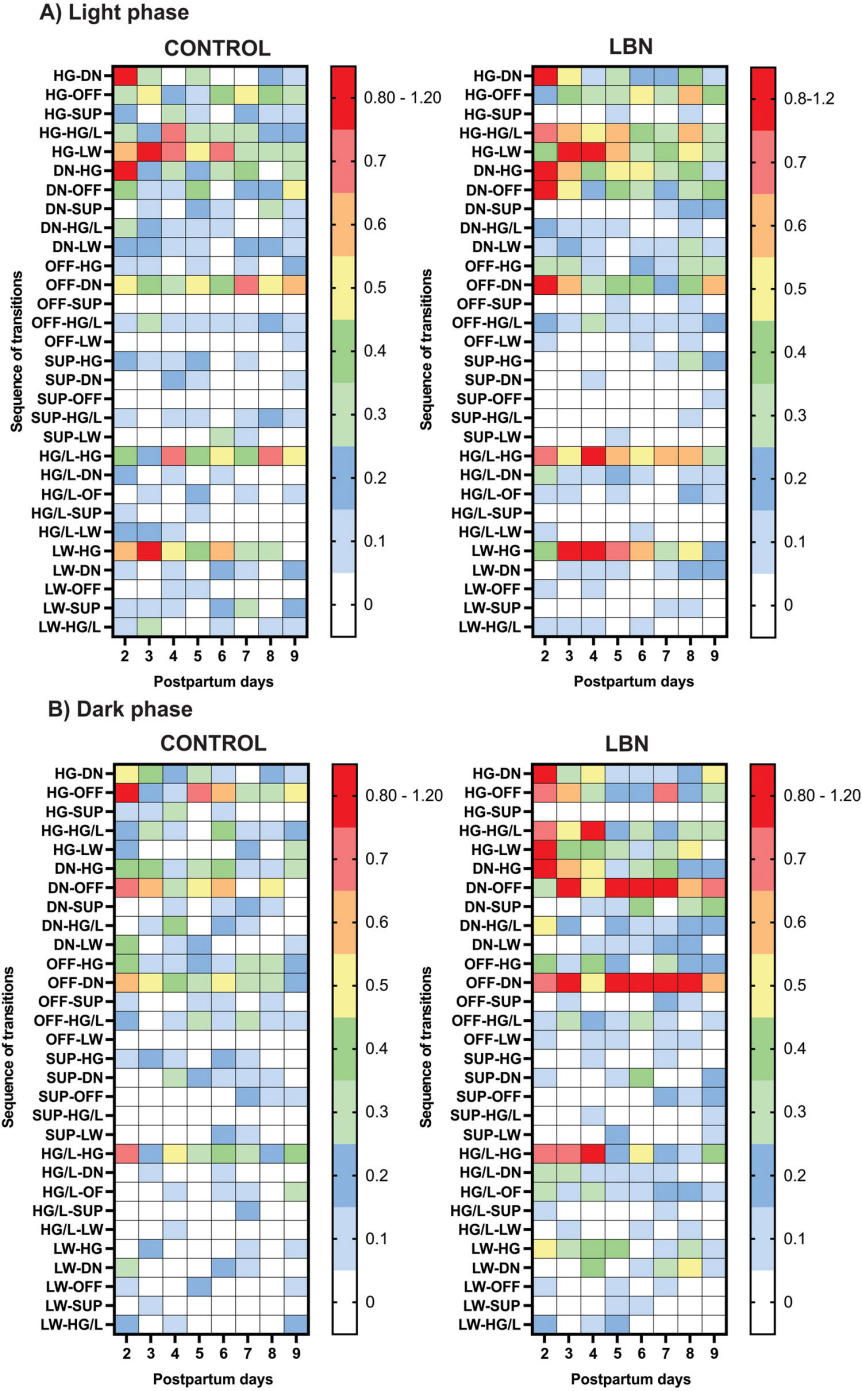
Heat map of resuming the behavioral transition in control and LBN networks from PPD 2 to PPD 9. Control and LBN maternal behavior transition heat map during the light (A) and dark phases (B). The graph of each map was generated with the weight of each transition. Transitions with the highest weight are depicted in red and the lowest in white, whereas the rest of the color range depicts transitions from low to high weight. DN, dam on the nest; HG, high crouch nursing posture; HG/L, high crouch nursing posture and licking pups; LW, low crouch nursing posture; OFF, dam off nest; SUP, supine nursing posture.

These results indicate that during the light and dark phases, the transition pattern differences between control and LBN were more evident during the early PPDs, summarized in the heat map graph (Figure 5). For example, during the light phase from PPD 2 to 5, control dams transitioned mostly from HG to LW and DN components but less to HG/L, whereas LBN dams transitioned mostly from HG to HG/L, LW, and DN components. On the other hand, from PPD 6 to 9, control dams mainly transitioned from OFF to DN and from HG/L to HG, whereas LBN dams transitioned mostly from HG to HG/L and from HG/L to HG (Figure 5A). Similarly, from PPD 2 to 4 of the dark phase, control dams transitioned more often from HG to OFF, whereas LBN dams transitioned from HG to HG/L, OFF, DN, and LW. From PPD 5 onwards, control dams transitioned more frequently from HG to OFF and DN to OFF, whereas LBN dams transitioned more frequently from DN to OFF and OFF to DN (Figure 5A).

## 4 Discussion

Here, we exposed rat dams and their pups to the LBN protocol from PPD 2 to 9 to investigate how it affects maternal behavior, specifically looking at how patterns of maternal behavioral transitions are changed. Behavioral transition analysis involves identifying specific components of maternal behavior that are crucial for understanding its effects on pup development. Through network analysis, we identified the components of maternal behavior that were most affected by LBN. Our results showed that the maternal behavior and behavioral transition patterns were altered under LBN conditions, particularly during the dark phase of observation. Network analysis revealed specific behavioral components that changed the pattern of behavioral transitions under the LBN.

### 4.1 Effects of LBN on Maternal Behavior

One of the most significant results was increased high crouch (HG) nursing posture events in LBN mothers. This increase was observed in the light and dark phases throughout the PPDs, with the dark phase profile very similar to the light phase day profile. This differs from what was observed in control dams, where the number of events in the dark phase was visibly reduced compared to the light phase. Previous literature also showed increased HG events in LBN dams (Dalle Molle et al. 2012; Eck et al. 2020; Granata et al. 2021; McLaughlin et al. 2016; Fuentes et al. 2014; Fuentes et al. 2018; Guadagno, Wong, and Walker 2018), indicating that this is a consistent alteration in maternal behavior of rats under LBN condition. During the light phase, increased HG behavior in LBN dams was accompanied by a significant reduction in supine (SUP) nursing posture and no change in behavioral transitions. In contrast, during the dark phase, it was accompanied by a significant decrease in OFF‐nest events and a significant increase in behavioral transitions. These findings suggest that LBN dams adjust their behavior differently during light and dark phases.

The mother–infant pair develops in a synchronized manner, adjusting their response to each other's input (Rosenblatt 1969). It is important to consider that the adjustment of the dam's behavior in LBN conditions depends in part on their pup's stimulation and the implications of this adjustment for their pup's development. The display of HG and LW nursing postures in rat mothers, characterized by immobility, is stimulated by warm pups that are actively rooting under her ventrum and attaching and sucking her nipples, where the pups can easily access milk flow (Stern and Johnson 1990). If the pups interrupt or do not successfully stimulate the mother, she may not maintain the nursing posture, causing interruptions. This could result in pups not receiving enough milk, leading them to continue stimulating the mother, even when she is engaged in non‐maternal activities outside the nest. During behavior observation, we noted that some LBN pups have their front or forelegs caught in the mesh during nursing events, particularly the youngest ones, who struggle to free themselves. This could contribute to infants interrupting maternal HG stimulation in the LBN condition. Future studies using the LBN paradigm should address this issue and explore alternatives to reduce pups caught in the mesh. One possible solution could be to place stable platforms without mesh in certain parts of the home‐cage.

Another explanation for the increase in HG behavior in LBN dams could be the huddling behavior of pups. Different from what we have observed in BALB/c and C57BL/N mice (Pardo et al. 2023), Sprague Dawley rats did not build a fixed nest using their fecal boli; they cut in pieces the paper towel or used it without cutting to cover the pups. This nest does not have limits to contain the pup's exits, so they continuously tend to move toward the mother and huddle near her, where she gathers the pieces of paper towel and arranges around them. Pups near the mother could have easily reached her ventrum and stimulated her HG behavior. Since the mother interacting with the huddling pups was considered an event of the mother in the nest, this may also explain why the OFF‐nest events are significantly reduced in the dark phase in LBN dams.

Our findings differ from those in the literature (Ivy et al. 2008; Molet et al. 2016; Alteba et al. 2016). Contrary to previous observations, we did not find licking and grooming pups' events in LBN different from control dams, which also has been observed by other LBN studies in rats (Fuentes et al. 2018; Guadagno, Wong, and Walker 2018). In fact, all dams in both groups exhibited this behavior at a very low level in all observation periods. Nest building and pup retrieval behaviors increased in LBN dams only on the 1st day of the LBN protocol, which might reflect the dams' habituation process to the new environment. We did not observe significant changes in pup‐off events in LBN litters. However, other studies have found that pups from LBN litters spend more time away from the nest (Brunson et al. 2005; Ivy et al. 2008; Moussaoui et al. 2016).

### 4.2 Effects of LBN on Pattern of Maternal Behavior Transitions

We have shown that LBN mothers showed increased behavioral transition between different behaviors in the dark phase but not during the light phase. We used network analysis to visualize these alterations and capture the changes between behavioral components that characterized the transition pattern of maternal behavior in the LBN condition. The behavioral transition in the LBN condition is an index of the fragmentation or inconsistency of maternal care under the hypothesis that consistent maternal care provides a predictable maternal sensory signal that influences neural circuits in the developing brain of pups (Baram et al. 2012). It is, therefore, important to identify the specific behavioral components that the LBN mothers transitioned most to provide insights into the types of sensory input the infants may be under or overexposed during early postnatal days.

The most significant result of network analysis was a distinct contrast between the behavior of LBN dams and control dams during the light and dark phases. During the light phase, both groups displayed similar transitioning patterns over the first four days of the LBN protocol, from HG to LW and DN and transitioning back to HG from all these components, with a difference that LBN dams also transitioned from HG to HG/L and from HG/L to HG, which were not observed in control dams. Nevertheless, LBN dams exhibited a different transition pattern during the dark phase, moving from HG to HG/L, OFF, DN, and LW and transitioning back to HG from all these components, whereas control dams only transitioned from HG to OFF, from DN to OFF, and from OFF to DN. In the last four days of the LBN protocol, LBN dams continued to transition from HG to HG/L during the light phase but also showed new transitions, including from LW to HG and from DN to LW. On the other hand, control dams mainly transitioned from OFF to DN and from HG/L to HG. During the dark phase, LBN dams mainly transitioned from DN to OFF and from OFF to DN, whereas control dams transitioned from HG or DN to OFF.

Mother rats display different caregiving behavior patterns during the first PPDs in light and dark phases. During the light phase, the mother spends most of her time in the nest caring for her pups, whereas, during the dark phase, she spends less time in the nest and more time outside, exhibiting non‐maternal behaviors. Network analysis has revealed that the maternal behavior pattern is uniquely altered or fragmented under light and dark phases under LBN conditions during early PPDs. In the light phase, the HG nursing posture is altered, whereas in the dark phase, the exits of the nest are disturbed. The HG component characterized by a posture of immobility of the mother (Rees, Lovic, and Fleming 2004) is frequently interrupted in LBN dams by engaging in licking pups at the same time she nurses them or by adopting a low back arched posture and returning frequently to HG from these postures. During the dark phase, exits from the nest and less contact with pups (DN) are often interrupted by the mother returning to the nest and engaging in nursing behaviors. A topic open for discussion is what causes the mother to disrupt her HG posture during the light phase and continually return to caring for pups during the dark phase under LBN conditions. Maternal behavior is synchronized with the behavioral development of infants, and their behavior is influenced by their interaction with them in each postpartum period (Rosenblatt 1969). One possible explanation could be related, at least in part, to factors affecting the pups' behavior, as discussed in the previous section.

During the final days of the postpartum period, LBN mothers continued to transition between nursing postures during the light phase, whereas control mothers increasingly left the nest area. Moreover, during the dark phase, LBN mothers persisted in interrupting their exits from the nest by frequently returning to be near their pups without nursing them.

### 4.3 Significance for Pups' Development

The timing of alteration of specific maternal care components under LBN conditions has profound implications for the pup's development. When nursing in a high crouch posture, the mother not only provides milk but also contributes to the sensory environment of the pups by exposing them to intense tactile, thermal, and olfactory stimulation. Continuous disruption of this behavior component in LBN dams during the early developmental period of their pups can affect the ongoing maturation of experience‐dependent systems with these sensory cues. Therefore, in addition to assessing the weight of the pups at the end of the LBN protocol to determine its impact on their nutritional status, it would also be important to evaluate in future studies the pace at which the infant developmental milestones, locomotor activity, and behavior progress and in parallel to explore the changes at the level of associated neural circuits.

### 4.4 Limitation of the Present Study

Our method of observing the dark phase represents only one observation period at the beginning of this phase, which may not capture the full pattern of maternal care exhibited during the night period. However, our data shows statistically significant differences between LBN and control mothers in the dark phase that are not observed in the light phase, suggesting that our findings are representative of the mothers' behavioral profile. Another limitation is that the network analysis provides an average profile of transitions of LBN and control mothers throughout the postpartum period based on nine mothers in each group, which does not capture the variations in the care strategy within each group. Future studies could address this limitation by analyzing the individual network for each mother rat's behavior.

## 5 Conclusion

In summary, exposure to LBN protocol from PPDs 2 to 9 led to alterations in maternal behavior by increasing high crouch nursing posture and behavioral transitions, which are more pronounced during the dark phase. In addition to detecting those changes, network analysis allowed us to capture the specific changes in those behavioral transitions, revealing patterns of predominant transition sequences between active nursing postures during the first four days of the LBN protocol, indicating that those components of maternal behavior are the most disrupted during this time window. The timing of these alterations has significant consequences for the ongoing maturation of infant systems that depend on the sensory experiences provided by the mother during nursing, which may help to explain the short‐ and long‐term behavioral and neurodevelopmental outcomes attributed to this paradigm.

## Author Contributions

**Grace E. Pardo**: conceptualization, data curation, formal analysis, visualization, writing–original draft, methodology, investigation, supervision, project administration, writing–review and editing, funding acquisition, resources. **Lucero B. Cuevas**: visualization, investigation, software. **Luis F. Pacheco‐Otalora**: resources, writing–review and editing. **Enver M. Oruro**: conceptualization, methodology, supervision, writing–review and editing, software, validation, resources, formal analysis, visualization.

### Peer Review

The peer review history for this article is available at https://publons.com/publon/10.1002/brb3.70113.

## Supporting information




**Table S1**. Summary of statistical analysis of non‐nursing dam behaviors
**Table S2**. Summary of intra‐group cumulative maternal behavior data comparison during LBN 2–9
**Figure S1**. Examples of network construction and visualization.
**Figure S2**. Non‐nursing maternal behavior profile during LBN protocol

## Data Availability

The data that support the findings of this study are available from the corresponding author upon reasonable request.
