# Supplementary material for: Altered Patterns of Maternal Behavior Transitions in Rats Exposed to Limited Bedding and Nesting Material Paradigm
Source: Brain Behav. 2024 Oct 23;14(10):e70113. doi: 10.1002/brb3.70113 (PMC11499211; doi:10.1002/brb3.70113)
Supplement: Supplementary file 1 — Table S1. Summary of statistical analysis of non‐nursing dam behaviors Table S2. Summary of intra‐group cumulative maternal behavior data comparison during LBN 2–9 Figure S1. Examples of network construction and visualization. Figure S2. Non‐nursing maternal behavior profile during LBN protocol [file BRB3-14-e70113-s001.docx]

**SUPPLEMENTARY MATERIAL**

**Table S1.**

Summary of Two-way repeated measure ANOVA results on dam behaviors as a function of postpartum day and housing condition (control and LBN).

|  | Factors | | | | |  | Interaction | |
| --- | --- | --- | --- | --- | --- | --- | --- | --- |
| **Parameters** | Postpartum day | |  | Housing | |  | Postpartum day x housing | |
| **Light phase** | ***F* (7,112)** | ***p*** |  | ***F* (1,16)** | ***p*** |  | ***F* (7,112)** | ***p*** |
| Dam on nest | 3.988 | **0.0006** |  | 1.121 | 0.3054 |  | 0.519 | 0.8187 |
| Dam off nest | 6.851 | **<0.0001** |  | 0.696 | 0.4163 |  | 0.999 | 0.4352 |
| Pup off | 0.608 | 0.7484 |  | 3.038 | 0.1005 |  | 0.815 | 0.5754 |
| Pup retrieval | 5.694 | **<0.0001** |  | 6.125 | **0.0249** |  | 2.906 | **0.0079** |
| Nest building | 23.26 | **<0.0001** |  | 3.197 | 0.0927 |  | 5.061 | **<0.0001** |
| Self-grooming | 0.545 | 0.7987 |  | 0.006 | 0.9371 |  | 1.463 | 0.1878 |
| **Dark phase** | ***F* (7,112)** | ***p*** |  | ***F* (1,16)** | ***p*** |  | ***F* (7,112)** | ***p*** |
| Dam on nest | 1.251 | 0.2813 |  | 1.577 | 0.2273 |  | 0.951 | 0.4710 |
| Dam off nest | 3.587 | **0.0016** |  | 24.43 | **0.0001** |  | 0.815 | 0.5765 |
| Pup off | 2.941 | 0.0073 |  | 0.5003 | 0.4895 |  | 1.032 | 0.4127 |
| Pup retrieval | 1.477 | 0.1824 |  | 4.397 | 0.0522 |  | 0.629 | 0.7313 |
| Nest building | 3.611 | **0.0015** |  | 12.41 | **0.0028** |  | 2.091 | 0.0501 |
| Self-grooming | 1.597 | 0.3501 |  | 0.741 | 0.4020 |  | 1.129 | 0.3501 |

Table showing Two-way RM ANOVA results for analysis of maternal behavior parameters with postpartum days and housing condition (control and LBN) as main factors. Bold text denotes *p*<0.05.


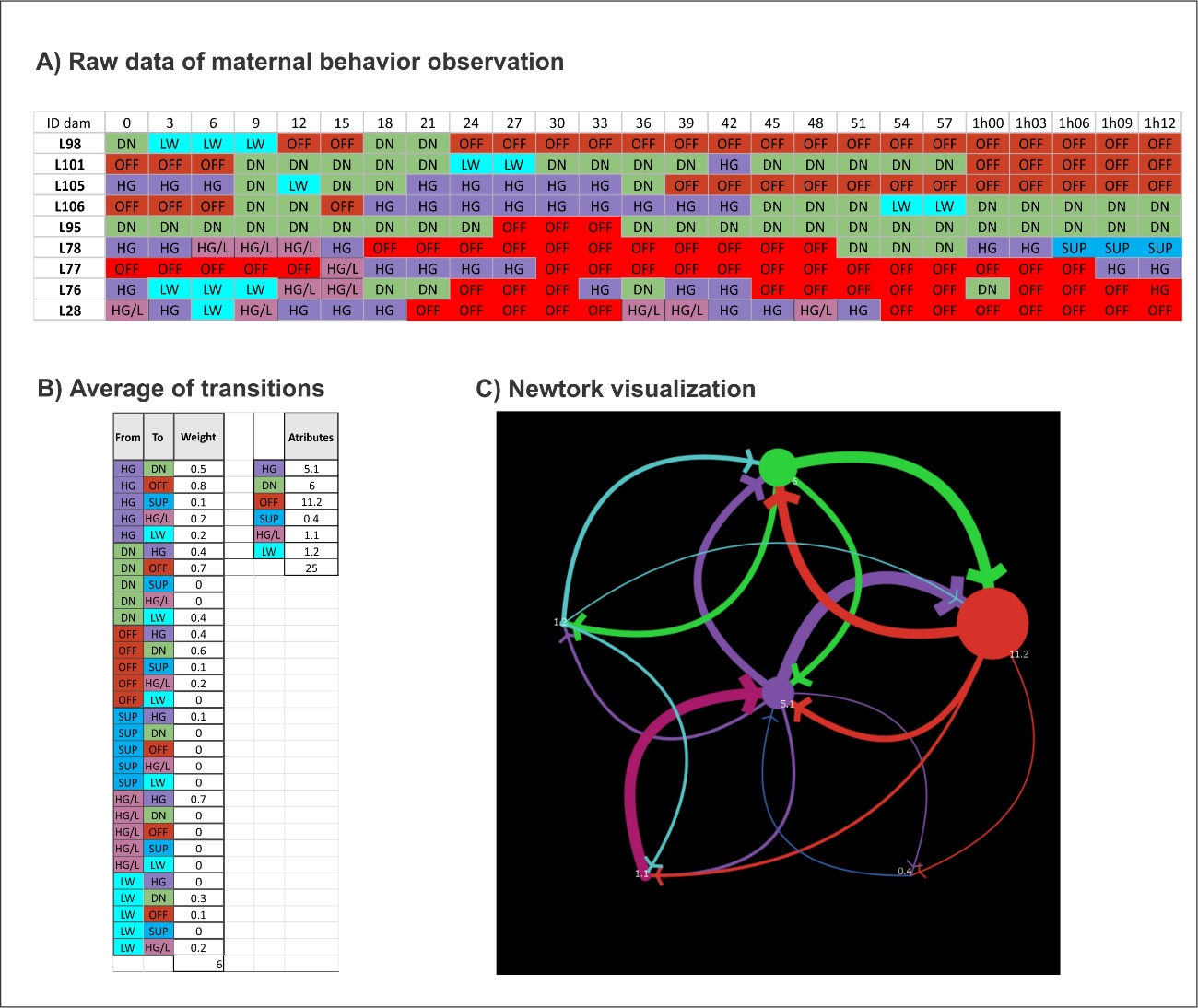


**Figure S1. Examples of network construction and visualization. A) Raw data of maternal behavior observation of 10 rats recorded during one observation period. Each maternal component is represented in a color code. B) The average of data in B represents the way how transitions between each behavioral component were calculated (weight) as well the frequency of each behavioral component (attributes), from which will be generated the thickness of the links and the size of the nodes. C) The network visualization graph was generated from the data in B. The links with arrows represent transitions from one behavioral component to another, and the ticker links indicate more frequent transitions. The sizer nodes in the network (red and green) indicate the more frequent components in that observation period. Networks were generated in NetLogo.**


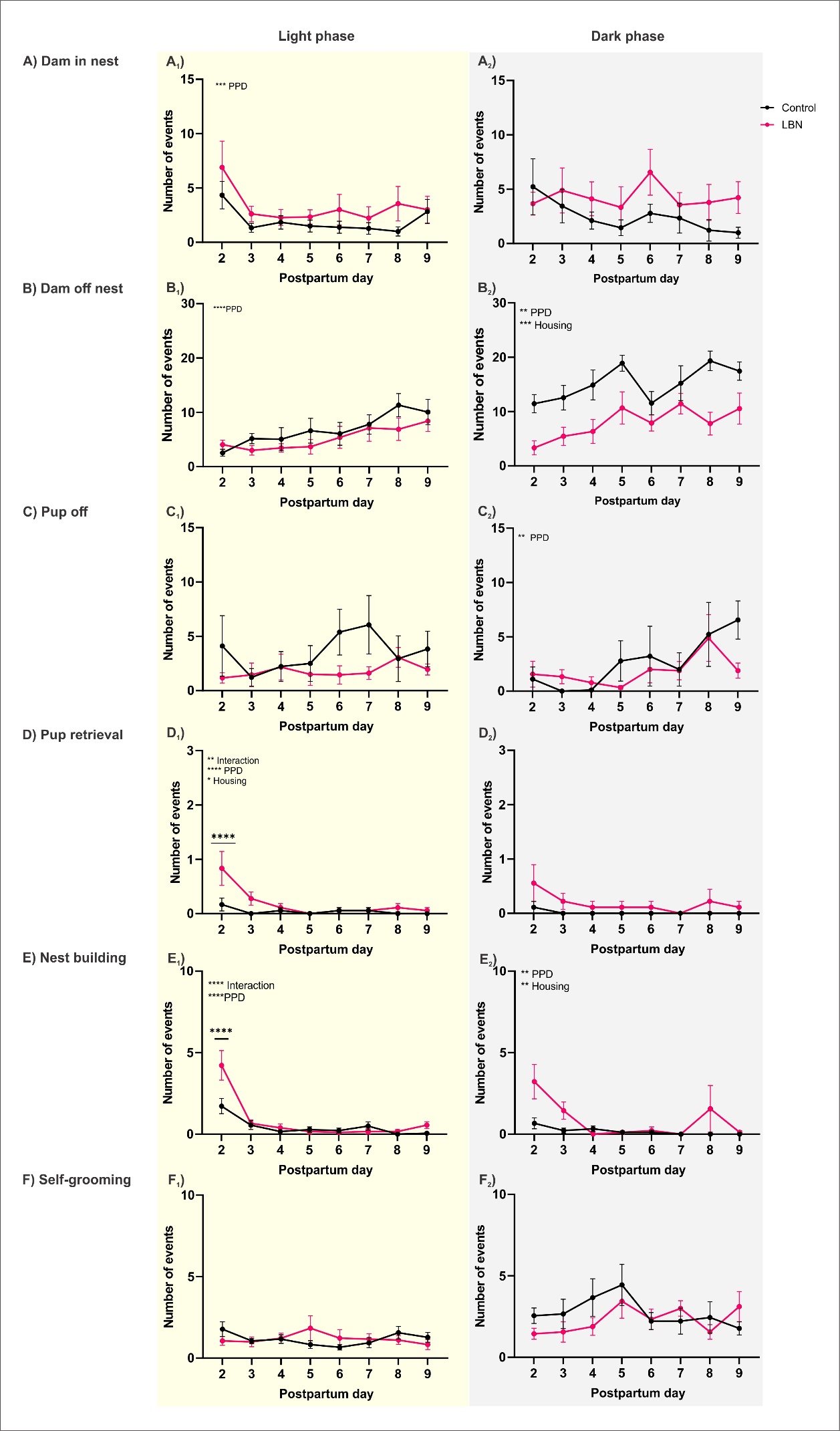


**Figure S2. Non-nursing maternal behavior profile during LBN protocol. D**am in nest (**A**) reduced significantly as a function of postpartum in light (**A1**) with no time-dependent changes during dark phase (**A2**). Dam off nest (**B**) did show changes along the postpartum days in light phase (**B1**) and also changes over the days in dark phase and it was affected by LBN (**B2**). Pup off (**C**) was not change along the postpartum days or LBN during the light phase (**C1**) but increased as a function of postpartum in the dark phase (**C2**). Pup retrieval (**D**) showed a significant interaction between postpartum days and LBN during the phase (**D1**) but not in the dark phase (**D2**). Nest building (**E**) showed a significant interaction between postpartum days and LBN during the phase (**E1**), which in the dark phase has independent main effect of both, where LBN showed an increased over control along the postpartum days (**D2**). Selfgroming (**F**) showed no effect of postpartum days nor LBN in light (**F1**) or dark phase (**F2**). Data are mean ± SEM. * p<0.05, **p<0.01, ***p<0.001, ****p<0.0001. N=9 per group.

**Table S2.**

**Summary of intra-group cumulative maternal behavior data comparison during LBN 2-9.**

|  | **Control** | | | |  | **LBN** | | | |
| --- | --- | --- | --- | --- | --- | --- | --- | --- | --- |
| **Light phase** | **9:00 AM** | **2:00 PM** | ***t (8)*** | ***p*** |  | **9:00 AM** | **2:00 PM** | ***t (8)*** | ***p*** |
| High crouch posture | 54.22±6.88 | 58.33 ±9.66 | 0.609 | 0.5595 |  | 70.44±7.74 | 106.7±7.28 | 3.408 | **0.0036** |
| Low crouch posture | 18.22±4.61 | 20.11±4.97 | 0.469 | 0.6520 |  | 15.00 ±3.12 | 20.56±4.94 | 1.437 | 0.1887 |
| Supine posture | 33.89±8.99 | 35.44±10.86 | 0.298 | 0.7730 |  | 12.78±7.16 | 8.333±2.30 | 0.654 | 0.5315 |
| High crouch posture & licking | 13.22±2.87 | 11.44±1.62 | 0.745 | 0.4774 |  | 10.67±3.41 | 12.11±2.25 | 0.617 | 0.5542 |
| Licking/grooming pups | 3.55±0.80 | 5.33±1.82 | 1.428 | 0.1911 |  | 6.667±1.25 | 4.333±1.05 | 3.101 | **0.0146** |
| Behavioral transitions | 47.22±4.46 | 50.44±4.04 | 1.181 | 0.2713 |  | 52.78±4.22 | 49.22±2.98 | 0.774 | 0.4613 |
| Dam on nest | 18.22± 5.06 | 21.89±5.41 | 1.713 | 0.1251 |  | 33.56±7.39 | 21.00±5.14 | 3.101 | **0.0015** |
| Dam off nest | 60.67±14.69 | 51.56±11.38 | 1.217 | 0.2581 |  | 56.44±11.97 | 29.44±5.60 | 3.685 | **0.0062** |
| Pup off | 33.67±7.60 | 32.11±8.022 | 0.217 | 0.8338 |  | 18.78±4.79 | 10.44±2.99 | 1.283 | 0.2354 |
| Pup retrieval | 0.444±0.34 | 0.222±0.15 | 0.800 | 0.4468 |  | 1.778±0.77 | 1.222±0.33 | 0.652 | 0.5325 |
| Nest building | 3.667±1.27 | 3.44±0.67 | 0.214 | 0.8358 |  | 7.667±1.43 | 5.222±1.56 | 1.350 | 0.2141 |
| Self-grooming | 9.778±2.26 | 10.11±1.02 | 0.136 | 0.8951 |  | 11.57±2.31 | 6.22±0.94 | 2.369 | **0.0453** |

The table shows the results of the paired Student t-test analysis comparing within-group data of maternal behavior accumulated at 9:00 a.m. and 2:00 p.m. for the control and LBN groups. Bold text indicates *p*<0.05.
